# Heterozygous *POLG* variant Ser1181Asn is associated with autosomal dominant neuro-myopathy in one family with no further specific manifestations of mitochondrial syndrome

**DOI:** 10.1186/s42466-022-00197-6

**Published:** 2022-07-11

**Authors:** Maike F. Dohrn, Danique Beijer, Lejla Mulahasanovic

**Affiliations:** 1 Department of Neurology, Medical Faculty, RWTH Aachen University, Aachen, Germany; 2 Dr. John T. Macdonald Foundation, Department of Human Genetics and John P. Hussman Institute for Human Genomics, University of Miami, Miller School of Medicine, Miami, FL USA; 3 Praxis für Humangenetik Tübingen, Tuebingen, Germany; 4 CeGaT GmbH, Tuebingen, Germany

## Comment

With great interest, we have read Finsterer et al.’s recent comment [1] on our article “Heterozygous *POLG* variant Ser1181Asn co-segregating in a family with autosomal dominant axonal neuropathy, proximal muscle fatigability, ptosis, and ragged red fibers” [2]. We appreciate the detailed discussion of our study’s strengths and weaknesses, the addressing of which gives us the opportunity to share more information on this interesting family where previously limited due to word count.

In our study, we described four affected family members, a father and three daughters, with a mixed neuro-myopathic phenotype, including features such as Charcot-Marie-Tooth disease-like axonal neuropathy and external ophthalmoplegia. Muscle biopsies revealed signs of mitochondrial structural abnormalities both on light and electron microscopy, and the so far undescribed heterozygous *POLG* variant c.3542G>A; p.Ser1181Asn in *POLG* (NM_001126131.3) co-segregated in all affected family members, whereas the unaffected sister (II.1) carried two wildtype alleles. Located within the enzyme’s palm domain that contains a cluster region of autosomal dominant variants, we hypothesized that this variant could well explain the family’s phenotype.

We are aware that this is an n of 1 study (case report), and that further replication and/or functional studies are needed to increase the genetic evidence further supporting the variant-disease relationship.

In their comment, Finsterer et al. suggest to analyze mitochondrial DNA for potential other mtDNA changes. Despite clear autosomal dominant inheritance from the father, mitochondrial DNA was assessed (NGS-based mitochondrial panel) in two individuals (II.2, II3), which did not reveal any relevant differential diagnoses. Of course, the screening for large indels or mitochondrial copy number variations was limited in regard to the methodological boundaries of whole-exome based diagnostics. A suggestion was made also to assess levels of mtDNA by rtPCR in order to show mitochondrial depletion as well as to conduct further biochemical analyses on muscle tissue homogenates. These are great suggestions that could additionally support the (clinical) mitochondrial phenotype already strengthened by COX-staining and electron microscopy.

It has further been recommended to perform a multisystemic assessment of other manifestations of mitochondrial disease, which we have actually done but so far not reported in detail. Cerebral MRIs performed in our proband (II.3, 2017) revealed unspecific, spotted, perivascular white matter lesions that did not show any clinical correlate. Patients II.2 and II.4 had normal MRIs of the neurocranium (II.2 in 2017, II.4 in 2011 and 2015). Cardiac evaluations including echocardiography (II.2, II.3, II.4), 24 h holter EKG (II.2, II.3, II.4), and cardiac MRI (II.3) did not show any signs of hypertrophic or dilated cardiomyopathy, nor signs of abnormal conductance. The patient-reported (II.4) proximal muscle fatiguability was assessed by detailed clinical examinations not showing any dynamics reminiscent of a myasthenic syndrome. Myasthenic antibodies such as anti-acetylcholine receptor antibodies were negative in patients II.3 and II.4. The same two sisters also underwent a spinal tap revealing normal CSF lactic acid, however, serum lactic acid was elevated (3.6 mmol/l [0.5–2.2]) in II.4 at one examination. Regarding other potential organ manifestations, patient II.4 had moderate sensorineural hearing loss confirmed by audiometry. None of the sisters showed signs of optic atrophy, however, II.3 developed a bilateral cataract in her mid 40 s.

In summary, we agree with Finsterer et al. that mitochondrial diseases merit a broad, systemic assessment, and that the pathogenicity of a single variant can be difficult to assess. Based on the patients’ phenotype and pedigree, the structural analyses of muscle tissue, as well as following the ACMG/ACGS-2020v4.01 guidelines for standardized classification of mendelian variants [3, 4], we still consider the variant p.Ser1181Asn in *POLG* likely pathogenic. Common obstacles for the interpretation of variant pathogenicity are incomplete penetrance, variability in age at onset and phenocopy rates, which have been taken into account here, using the most recent version of ACGA criteria [4]. We are happy to collaborate for further studies in this field.
